# *Centipeda minima* (*Ebushicao*) extract inhibits PI3K-Akt-mTOR signaling in nasopharyngeal carcinoma CNE-1 cells

**DOI:** 10.1186/s13020-015-0058-5

**Published:** 2015-09-18

**Authors:** Yu-qing Guo, Hai-yan Sun, Chi-on Chan, Bei-bei Liu, Jian-hong Wu, Shun-wan Chan, Daniel Kam-Wah Mok, Anfernee Kai-Wing Tse, Zhi-ling Yu, Si-bao Chen

**Affiliations:** Institute of Medicinal Plant Development, Chinese Academy of Medical Sciences and Peking Union Medical College, Beijing, 100193 People’s Republic of China; State Key Laboratory of Chinese Medicine and Molecular Pharmacology, Department of Applied Biology and Chemical Technology, The Hong Kong Polytechnic University, Shenzhen, 518057 People’s Republic of China; School of Chinese Medicine, Hong Kong Baptist University, Hong Kong, People’s Republic of China

## Abstract

**Background:**

*Centipeda minima* (*Ebushicao*) has been used for the treatment of various diseases, such as nasal allergies, rhinitis and sinusitis, nasopharyngeal carcinoma, cough, and headache. This study aims to investigate the anticancer activities of *Centipeda minima* ethanol extracts (CME) against nasopharyngeal carcinoma cell CNE-1 and their underlying mechanism.

**Methods:**

CNE-1 cells were treated with different concentrations (15–50 μg/mL) of CME for different time intervals (24, 48, and 72 h). Cytotoxicity of CME was determined by MTT assay. Cell morphological changes were observed by fluorescence microscopy after HO 33258 staining. Cell cycle status was evaluated by flow cytometry following propidium iodide staining. Apoptosis was detected by flow cytometry following annexin V-FITC/PI staining. The levels of apoptosis-associated and PI3K-Akt-mTOR signaling related proteins were measured by western blotting analysis.

**Results:**

CME (15–50 μg/mL) significantly inhibited the proliferation of CNE-1 in a dose- and time-dependent manner (*P* = 0.026 for 15 μg/mL, *P* < 0.001 for 25, 30, 40, and 50 μg/mL, respectively); the IC_50_ values (μg/mL) were 41.57 ± 0.17, 30.34 ± 0.06 and 24.98 ± 0.08 for 24, 48 and 72 h treatments, respectively. Significant morphological changes of CNE-1 cells displaying apoptosis were observed after CME treatment. CME showed low cytotoxicity toward normal LO2 cells. CNE-1 cells were arrested in the G2/M phase while treated with 15, 25, 40 μg/mL of CME, respectively (*P* = 0.032, *P* = 0.0053, P < 0.001). CME (15, 25, 40 μg/mL) down-regulated Bcl-2 expression (*P* = 0.032, *P* = 0.0074, *P* < 0.001), and up-regulated Bax (*P* = 0.026, *P* = 0.0056, *P* < 0.001) with activation of caspase-3, caspase-8, caspase-9, and PARP observed in CNE-1 cells (*P* = 0.015, *P* = 0.0067, *P* < 0.001 for caspase 3; *P* = 0.210, 0.028, < 0.001 for caspase 8; *P* = 0.152, 0.082, 0.0080 for caspase 9; *P* = 0.265, 0.0072, < 0.001 for PARP). CME suppressed the activation of the PI3K-AKT-mTOR pathway (*P* = 0.03, 0.0007, 0.004, 0.006, 0.022 for p-PI3K, p-Akt-Ser^473^, p-Akt-Thr^308^, p-mTOR-Ser^2448^, p-mTOR-Ser^2481^, respectively after 40 μg/mL of CME treated for 24 h).

**Conclusion:**

CME inhibited the proliferation of CNE-1 cells and activation of the PI3K-AKT-mTOR signaling pathway.

## Background

Nasopharyngeal carcinoma (NPC), a malignant epithelial tumor derived from the nasopharyngeal surface epithelium, has a low incidence in Western countries but a high incidence in Southeast Asia, southern China and the Mediterranean basin [1]. Epstein-Barr virus infection is a critical factor in NPC pathogenesis [2, 3]. Additional etiological factors for NPC include genetic susceptibility, environmental contamination, and dietary habits such as excessive consumption of salt-preserved fish during childhood [1, 4]. Radiotherapy is the standard primary treatment for NPC [5], and a combination of radiotherapy and chemotherapy has been shown to be an effective therapeutic strategy for treating advanced NPC [6]. The most prevalent chemotherapeutic drugs used to treat NPC are cisplatin-based drugs, which are highly toxic and prone to drug resistance [7, 8]. Chinese medicine (CM) in combination with conventional cancer therapies has been applied to NPC [9]. Controlled clinic trials demonstrated that CM therapies achieve prolonged survival and improved quality of life in NPC patients [10, 11].

Some CMs exhibit antitumor activities toward NPC cells via growth inhibition, apoptotic induction and cell cycle arrest [12, 13]. *Centipeda minima* (L.) A. et Aschers. (*Ebushicao*) (Asteraceae) has been used to treat nasal allergies, rhinitis, sinusitis, cough, headache [14, 15] and NPC [16]. Recent researches have focused on its anti-NPC activities, leading to the isolation of a few sesquiterpene lactones against NPC cells [17, 18]. Volatile oils from *C. minima* extracted by steam distillation and supercritical fluid extraction induced CNE cell death via induction of intrinsic apoptosis by regulating the expression of the Bcl-2 family of proteins [16]. Ethyl acetate extracts of *C. minima* exhibited anti-proliferative activity against CNE-2 cells [19]. However, the anticancer activities of *C. minima* ethanol extracts (CME) against the NPC cell line CNE-1, and their underlying mechanism, remain unclear.

This study aims to investigate the anticancer activities of CME against the nasopharyngeal carcinoma cell line CNE-1 and determine their underlying mechanism.

## Methods

### Plant materials and sample preparation

*Centipeda minima* was collected in August 2010 in Xiangfan, Hubei Province, China (latitude, 32°04′ N; longitude, 112°05′ E), and authenticated by Si-bao Chen based on morphological features. A voucher specimen (EBSC-016-09) was deposited at the herbarium of the State Key Laboratory of Chinese Medicine and Molecular Pharmacology, Department of Applied Biology and Chemical Technology, The Hong Kong Polytechnic University. The herb was air-dried and ground to coarse powder. The powder (30 g) was macerated with 0.5 L of 95 % ethanol at room temperature for 72 h. The extraction was repeated twice. After extraction, the ethanol extracts were combined, filtered through Whatman filter paper (Whatman, Maidstone, UK), and evaporated to dryness using a rotary evaporator (Precision MLG3, Heidolph, Germany) on a water bath at 40 °C, and further lyophilized to dried powder (3.80 g). CME samples with a yield of 12.65 % were stored in the refrigerator at 4 °C until further use. CME was dissolved in DMSO and filtered to 0.22 μm to obtain the stock concentration of 20 mg/mL. Finally, a serial dilution to concentrations of 15, 25, 30, 40, and 50 μg/mL CME was performed. DMSO-treated cells were employed as a vehicle control in all experiments.

### Chemicals

3-(4,5-Dimethylthiazole-2-yl)-2,5-diphenyltetrazolium bromide (MTT), dimethyl sulfoxide (DMSO), Hoechst 33258 and cisplatin were obtained from Sigma (St. Louis, MO, USA). The annexin V-FITC apoptosis detection kit and cell cycle analysis kit were purchased from Beyotime (Invitrogen, Carlsbad, CA, USA). Precision Plus Protein Standards (Dual Color) and Immun-Star™ WesternC™ Chemiluminescent Kit were purchased from Bio-Rad (Hercules, CA, USA). Antibodies against procaspase 8, cleaved caspase 9, cleaved PARP, PI3K p110α, Akt, p-Akt (Thr^308^ and Ser^473^), mTOR and p-mTOR (Ser^2448^ and Ser^2481^) were obtained from Cell Signaling Technology (Beverly, MA, USA). All other primary antibodies, as well as anti-rabbit and anti-mouse secondary horseradish peroxidase antibodies, were purchased from Abcam (Cambridge, MA, USA). All other common chemicals were reagent grade.

### Cell culture

CNE-1 human nasopharyngeal carcinoma cells (depository no. CBP60002) were purchased from the Cancer Institute and Hospital, Chinese Academy of Medical Sciences (Beijing, China). LO2 human normal liver cells (depository no. GNHu 6) were purchased from the Cell Bank of Chinese Academy of Sciences (Shanghai, China). Cells were cultured in RPMI-1640 medium (Invitrogen, USA) supplemented with 10 % fetal bovine serum (Gibco, Life Technologies, Grand Island, NY, USA), 100 IU/mL penicillin, 100 μg/mL streptomycin (Thermo Fisher Scientific, Madison, WI, USA) at 37 °C in a humidified incubator with a 5 % CO_2_ atmosphere. The medium was renewed three times a week. Cells in logarithmic growth phase were used for all experiments.

### Cell viability assay

The effects of CME on the viability of CNE-1 and LO2 cells were evaluated by MTT assay [20]. Cells (3 × 10^4^ cells/mL) were seeded into the wells of 96-well culture plates (Thermo Fisher Scientific) in 100 μL of medium per well and then allowed to adhere for 24 h at 37 °C in a 5 % CO_2_ atmosphere. After incubation, the cells were treated with the target concentrations of CME (15–50 μg/mL), 0.1 % DMSO as the vehicle control, and cisplatin (4 μg/mL) as a positive control, for 24 h. After incubation for 24, 48, and 72 h at 37 °C in a CO_2_ incubator, 10 μL of MTT solution [5 mg/mL in phosphate-buffered saline (PBS)] was added to each well and incubated for a further 4 h. Then, excess medium was removed and 150 μL of DMSO was added to each well to dissolve the formazan crystals. The optical density in each well was measured using a microplate spectrophotometer (BMG POLARstar Galaxy, Offenburg, Germany) at 490 nm. Triplicate experiments were performed for treatment with each concentration. The percentage cell viability (%) was calculated by comparison with a sample’s corresponding control. IC_50_ values were calculated to evaluate the cytotoxic effects of treatments on CNE-1 cells.

### Morphological assessment

The cells (1 × 10^6^ cells/mL) were seeded into 6-well plates, and then treated with different concentrations (15, 25, 30, 40, or 50 μg/mL) of CME for 24 h. Similarly, cells were treated with 25 μg/mL CME for 24, 48, and 72 h, respectively. DMSO (0.1 %)-treated cells were employed as controls. Subsequently, the cells were washed twice with cold PBS, fixed with 4 % paraformaldehyde solution for 10 min, stained with Hoechst 33258 (10 μg/mL) for 15 min in the dark, and then observed under an inverted fluorescence microscope (Leica DMRB, Weitzl, Germany) at the excitation wavelength of 352 nm and emission wavelength of 461 nm.

### Apoptosis analysis by annexin V-FITC/PI staining

The numbers of apoptotic cells were assessed using an annexin V-FITC apoptosis detection kit (Invitrogen, USA) according to the manufacturer’s protocol. Cells (1 × 10^6^ cells/mL) were treated with various concentrations of CME for 24, 48, and 72 h. Cells were then harvested, and re-suspended in binding buffer. Subsequently, cells were stained with 5 mL of annexin V-FITC and 5 mL of propidium iodide (PI) for 15 min at room temperature in the dark. The apoptotic cell percentages were then immediately detected using a FACSCalibur flow cytometer (BD Biosciences, San Jose, CA, USA).

### Cell cycle analysis by PI staining

Cells (1 × 10^6^ cells/well) were treated with various concentrations of CME (15, 25, and 40 μg/mL) for 24, 48, and 72 h. Cells were then collected and washed twice with 1 mL of ice-cold PBS, suspended in 1 mL of ice-cold 70 % ethanol (containing 1 × 10^5^ cells) and kept overnight at 4 °C. The next day, cells were washed with PBS and incubated with 500 μL of PI staining solution (containing PI 40 μg/mL, RNase A 100 μg/mL and PBS) for 30 min in the dark at 37 °C. Then, samples were filtered using 70-μm filters into test tubes and detected by flow cytometry (FACSCalibur, Bio-Rad). Analysis of cell cycle status was performed using ModFit LT 3.0™ software (BD Biosciences).

### Western blot analysis

CNE-1 cells (1 × 10^6^ cells/well) were seeded into 100 mL culture dishes for 24 h and then treated with various concentrations of CME (15, 25, and 40 μg/mL). After incubation for 24 h, the cells were harvested and prepared in NP-40 lysis buffer (2 mM Tris–Cl pH 7.5, 150 mM NaCl, 10 % glycerol and 0.2 % NP-40 plus a protease inhibitor cocktail) for 30 min on ice. Cell lysates were ultra-centrifuged (Allegra™ 21R Centrifuge, Beckman Coulter, Krefeld, Germany) at 14,000×*g* for 10 min at 4 °C, and then the supernatant was collected as the total cellular proteins. Protein concentrations were determined using a BCA protein assay kit (Beijing ComWin Biotech Co.,Ltd., Beijing, China). Proteins (10 μg for β-actin and Bax, 20 μg for PARP and cleaved PARP, 30 μg for caspase-3, -9, and -8, PI3K p110α, Akt, and p-Akt, 50 μg for Bcl-2, mTOR, and p-mTOR) were resolved on 12–15 % SDS-PAGE gels, then transferred to PVDF (polyvinylidene difluoride) membranes (Roche Applied Science, Basel, Switzerland) and blocked with 5 % non-fat dry milk in Tris-buffered saline containing 0.1 % Tween-20 (T-TBS) overnight at 4 °C. After washing with T-TBS, the membranes were incubated with primary antibodies against procaspase-3, -8, and -9; caspase-3, -8, and -9; Bcl-2, Bax, PARP, cleaved PARP; PI3K p110α, Akt, p-Akt (Thr^308^ and Ser^473^), mTOR, p-mTOR (Ser^2448^ and Ser^2481^), and β-actin overnight at 4 °C. Subsequently, the membranes was incubated with goat anti-rabbit or horseradish peroxidase-conjugated goat anti-rabbit secondary antibodies (Merck Millipore) at room temperature for 2 h at dilutions of 1:5000 and 1:10,000, respectively. Antibody-bound proteins were detected, normalized to β-actin and quantified in a ChemiDoc™ XRS system (Universal Hood II, Bio-Rad) using an Immun-Star™ HRP Chemiluminescence Kit (Bio-Rad).

### Statistical analysis

All data are expressed as the mean ± standard deviation (SD) of at least three independent experiments. Analyses of dose-, concentration- and time-dependent effects were performed by sigmoidal non-linear regression using GraphPad Prism 5.02 software (GraphPad Software, Inc., La Jolla, CA, USA). The percentage cell viability (%) after CME treatment with different concentrations and time intervals was used for the fitting. The two-tailed Student’s *t* test was used for comparisons of two groups, and multi-group differences were analyzed by one-way analysis of variance (ANOVA) followed by Dunnett’s multiple comparison test. *P* values < 0.05, 0.01, or 0.001 were considered statistically significant.

## Results

### Cytotoxic effects of CME on CNE-1 cells

**Fig. 1:**
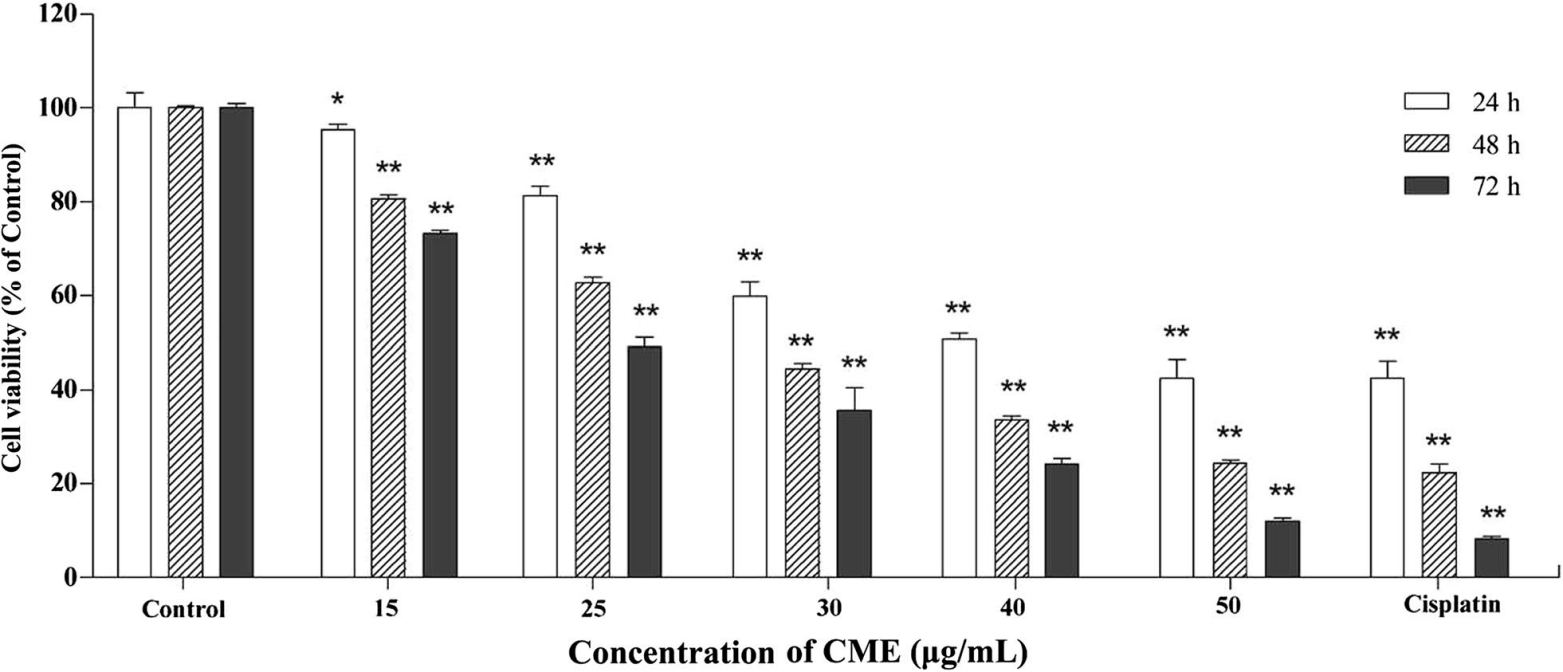
Dose- and time-dependent cytotoxic effects of CME on CNE-1 cells. CNE-1 cells were treated with 0.1 % DMSO (vehicle control), 4 μg/mL cisplatin (positive control) and various concentrations (15–50 μg/mL) of CME for 24, 48, and 72 h, respectively, and then cell viability was determined by MTT assay. Values are the mean ± SD of at least three independent experiments. *P < 0.05, **P < 0.001 versus control

CNE-1 cells were treated with various concentrations (15–50 μg/mL) of CME for 24, 48, and 72 h, and the cell viability was determined by MTT assay. CME decreased CNE-1 cell viability in a dose- and time-dependent manner (Fig. 1; Table 1). After treatment with CME (15, 25, 30, 40 and 50 μg/mL) for 24 h, the percentage of live cells were 95.41, 81.27, 60.10, 50.0 and 42.41 %, (*P* = 0.026 for 15 μg/mL, *P* < 0.001 for 25, 30, 40, and 50 μg/mL), respectively. Meanwhile, those were 51.14, 34.0, and 25.05 % (*P* < 0.001 for each test), while CNE-1 cells were treated with CME (40 μg/mL) for 24, 48, and 72 h, respectively. The IC_50_ values were 41.57 ± 0.17, 30.34 ± 0.06 and 24.98 ± 0.08 μg/mL for 24, 48, and 72 h incubations, respectively. Despite its potency in CNE-1 cells, CME exhibited no significant cytotoxic effect on normal LO2 cells (data not shown due to no significant variation versus vehicle control).

**Table 1:** The *P* value of significance analysis in each experiment on the cytotoxicity of CME against CNE-1 cells

Duration of treatment (h)	Concentration of CME (μg/mL)				
	15	25	30	40	50
24	0.032	0.0006	0.0005	0.0007	0.0006
48	0.0009	0.0008	0.0004	0.0005	0.0002
72	0.0007	0.0002	0.0006	0.0003	0.0001

### Morphological changes in CNE-1 cells after CME treatment

**Fig. 2:**
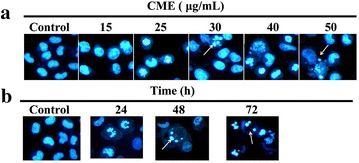
Cell morphological changes induced by CME. **a** Cells treated with various concentrations of CME (15, 25, 30, 40, and 50 μg/mL) for 24 h. **b** Cells treated with 25 μg/mL for 24, 48, and 72 h. The cells were fixed after CME treatment, stained with HO 33258, and their nuclear morphology was photographed under fluorescence using a blue filter (×400). The *arrow* points to an apoptotic body

Morphological changes and cell death in CNE-1 cells were observed under an inverted fluorescence microscope (DFC 420 C, Leica, Germany) after HO 33258 staining. Vehicle-treated (control) cells showed normal cell architecture with clear cytoskeletons (Fig. 2). Typical morphological changes associated with apoptosis, including chromatin condensation, apoptotic body formation and nuclear degradation, were observed in CME-treated cells. These morphological changes were enhanced with increasing concentrations of CME (Fig. 2a), and with increasing treatment duration (Fig. 2b).

### Apoptosis analysis by flow cytometry

**Fig. 3:**
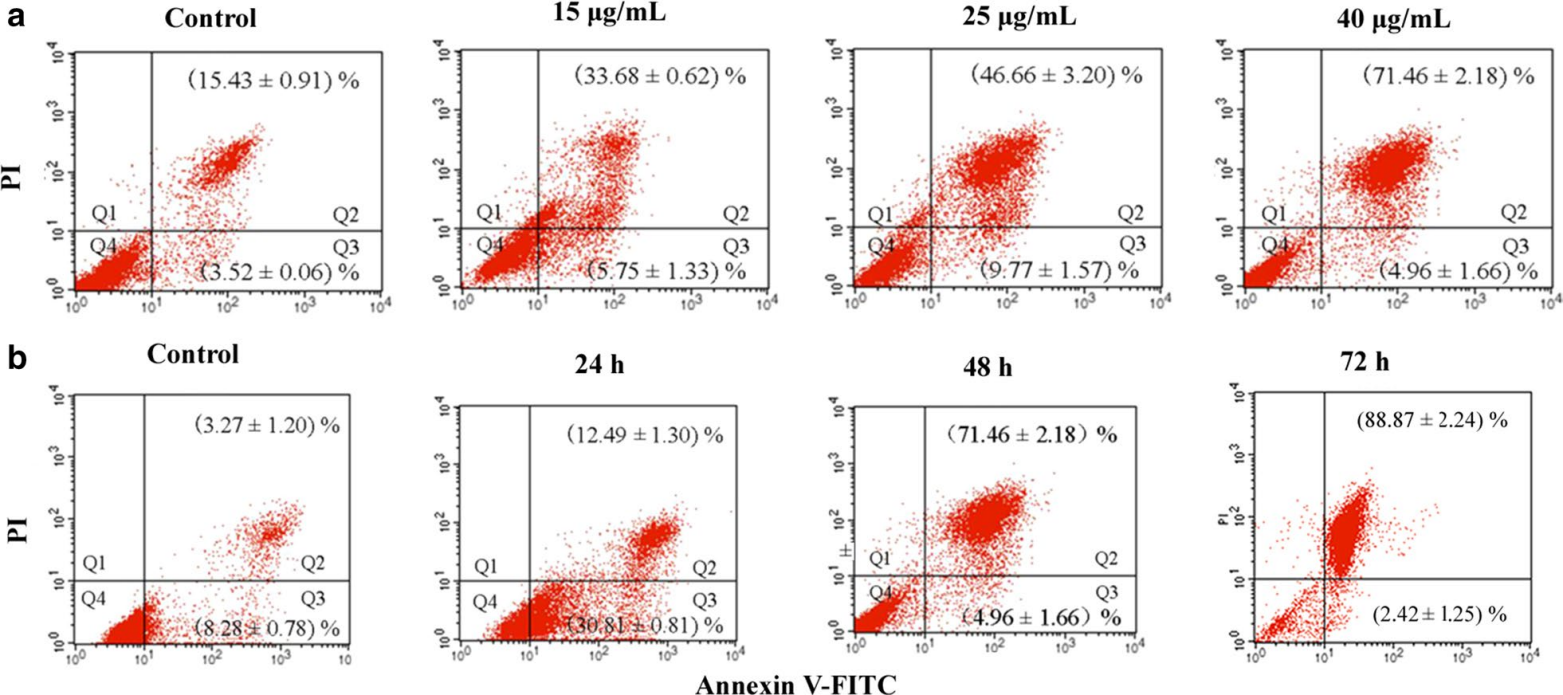
Flow cytometry analysis of the induction of apoptosis in CNE-1 cells by CME. Results are shown in logarithmic fluorescence intensity with the *x*-axis (annexin V-FITC) versus *y* axis (PI). Four quadrants represent necrotic cells (Q1: AV^−^/PI^+^), late apoptotic cells (Q2: AV^+^/PI^+^), early apoptotic cells (Q3: AV^+^/PI^−^) and viable cells (Q4: AV^−^/PI^−^). *Numbers* indicate the percentages of cells in each quadrant and a minimum of 5000 events were read (*n* = 3). **a** CNE-1 cells were treated with 15, 25, and 40 μg/mL CME for 48 h; **b** CNE-1 cells were treated with 40 μg/mL CME for 24, 48, and 72 h

The timing of induction of apoptosis was determined by flow cytometry following annexin V-FITC/PI double staining. After exposure to CME (15, 25, or 40 μg/mL) for 48 h, the proportion of apoptotic cells dramatically increased in a dose-dependent manner; the percentages of apoptotic cells were 39.53, 56.43, and 76.42 %, respectively (*P* = 0.0086, *P* < 0.001, *P* < 0.001) (Fig. 3a). Similarly, CME induced CNE-1 cell apoptosis in a time-dependent manner. The percentages of apoptotic cells were 43.30, 76.42, and 91.29 % after treatment with 40 μg/mL CME for 24, 48, and 72 h, respectively (*P* < 0.001 for each test) (Fig. 3b).

### Flow cytometric analysis of the cell cycle status of CNE-1 cells

**Fig. 4:**
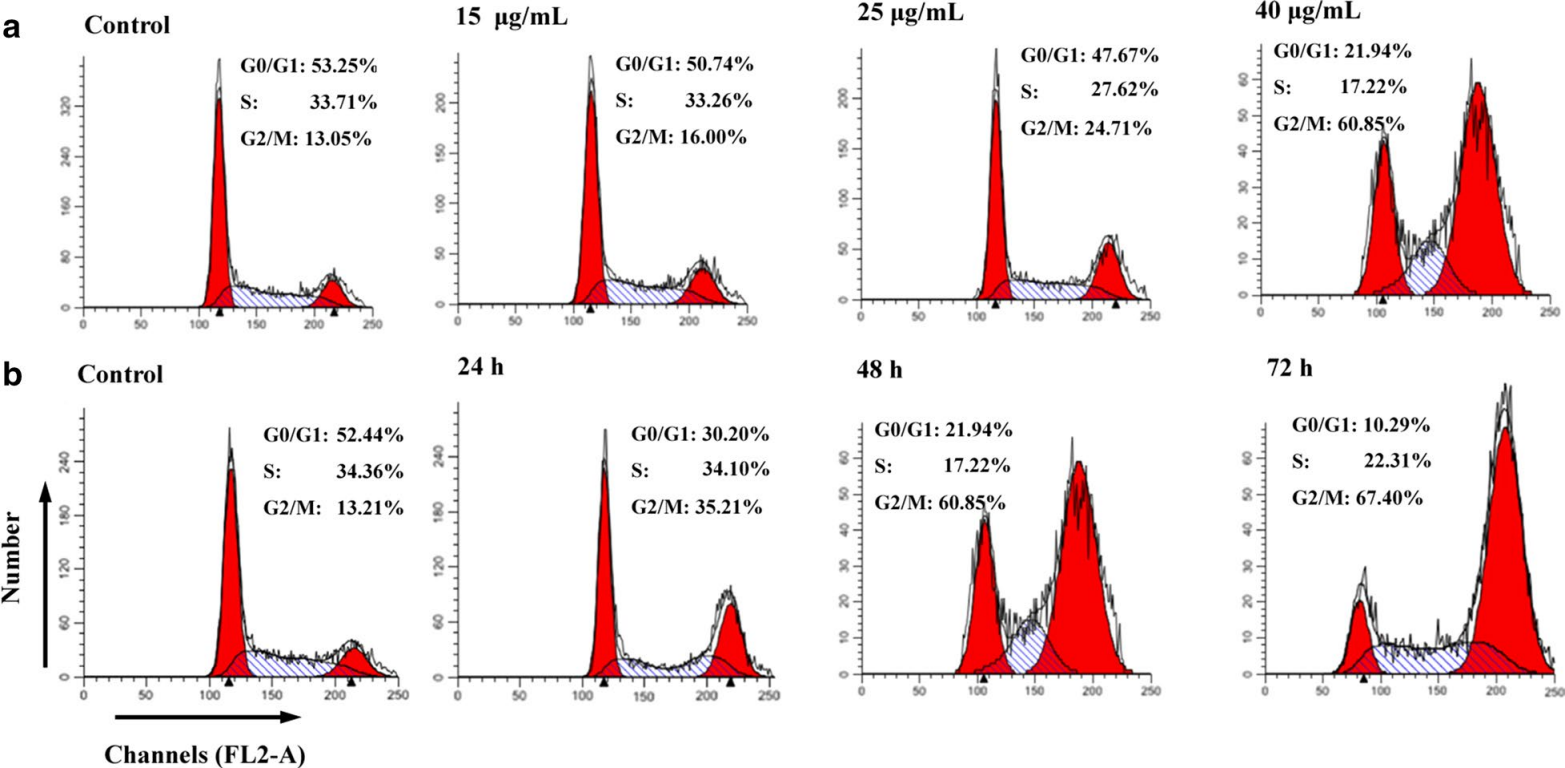
Cell cycle distribution of CNE-1 cells in the flow cytometric analysis after CME treatment. **a** CNE-1 cells were treated with 0.1 % DMSO (control) and 15, 25, and 40 μg/mL CME for 48 h; **b** CNE-1 cells were treated with 40 μg/mL CME for 0 (control), 24, 48, and 72 h

Cell cycle progression was analyzed by flow cytometry to determine whether the cytotoxic effect of CME toward CNE-1 cells was associated with the induction of cell cycle arrest. After treatment with 15, 25, and 40 μg/mL CME for 48 h, the percentages of cells in the G2/M phase were 16.00, 24.71, and 60.85 %, respectively (*P* = 0.032, *P* = 0.0052, *P* < 0.001); while the percentages of those in the G0/G1 phase were 50.74, 47.67, and 21.94 %, respectively (*P* = 0.102, 0.067, *P* < 0.001) (Fig. 4a). Meanwhile, after treatment with CME (40 μg/mL) for 24, 48, and 72 h, the percentages of cells in the G2/M phase were 29.38, 35.21, and 41.13 %, respectively (*P* < 0.001 in each test); while the percentages of those in the G0/G1 phase were 42.69, 30.20, and 25.64 %, respectively (*P* = 0.0072, *P* = 0.0047, *P* < 0.001) (Fig. 4b). No significant variations in the percentages of cells in S phase were observed after CME treatment. These results indicated that the inhibition of CNE-1 cell proliferation induced by CME could be attributed to cell cycle arrest in the G2/M phase.

### Expression of CME-regulated apoptosis-related proteins

**Fig. 5:**
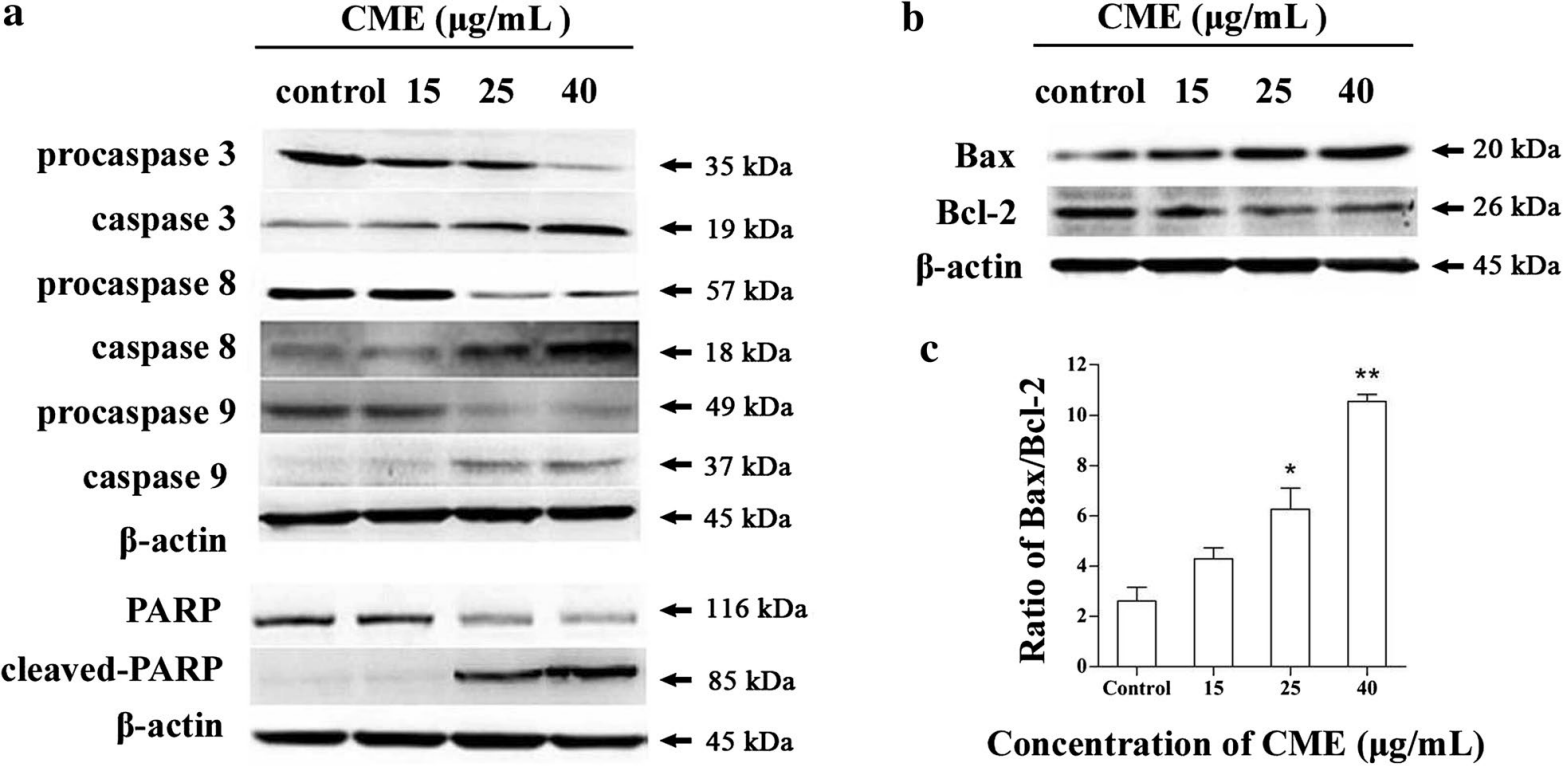
Effects of CME on the expression levels of apoptosis-related proteins in CNE-1 cells after 24 h of treatment. **a** Expression levels of procaspase-3, -8, and -9; caspase-3, -8, and -9; PARP and cleaved-PARP. **b** Expression level of Bcl-2 and Bax. **c** Bax/Bcl-2 ratios; each value is based on three independent experiments. *P = 0.034, **P = 0.0005 versus control

The expression levels of apoptosis-related proteins were analyzed by western blotting to study CME-induced apoptosis of CNE-1 cells in more detail. After treatment with CME (15, 25, 40 μg/mL) for 24 h, the levels of cleaved caspase 3, 8, 9 and cleaved PARP increased in a dose-dependent manner (*P* = 0.020, *P* = 0.0060, *P* < 0.001 for caspase 3; *P* = 0.210, 0.028, < 0.001 for caspase 8; *P* = 0.152, 0.082, 0.0082 for caspase 9; *P* = 0.265, *P* = 0.0060, *P* < 0.001 for PARP), while the levels of pro-caspases 3, 8, 9 and PARP decreased (*P* = 0.0045, *P* < 0.001, *P* < 0.001 for pro-caspase 3; *P* = 0.147, 0.015, 0.036 for pro-caspase 8; *P* = 0.250, 0.031, 0.0085 for pro-caspase 9; *P* = 0.092, < 0.001, 0.001 for pro-PARP) (Fig. 5a). Meanwhile, the anti-apoptotic protein Bcl-2 was down-regulated (*P* = 0.035, *P* = 0.0055, *P* < 0.001), while the pro-apoptotic protein Bax was up-regulated (*P* = 0.020, *P* = 0.0035, *P* < 0.001) (Fig. 5b), and the Bax/Bcl-2 ratio was significantly increased as the concentrations of CME increasing (*P* = 0.072, 0.034, 0.0005) (Fig. 5c).

### Inhibition of PI3K-Akt-mTOR signaling in CME-treated CNE-1 cells

**Fig. 6:**
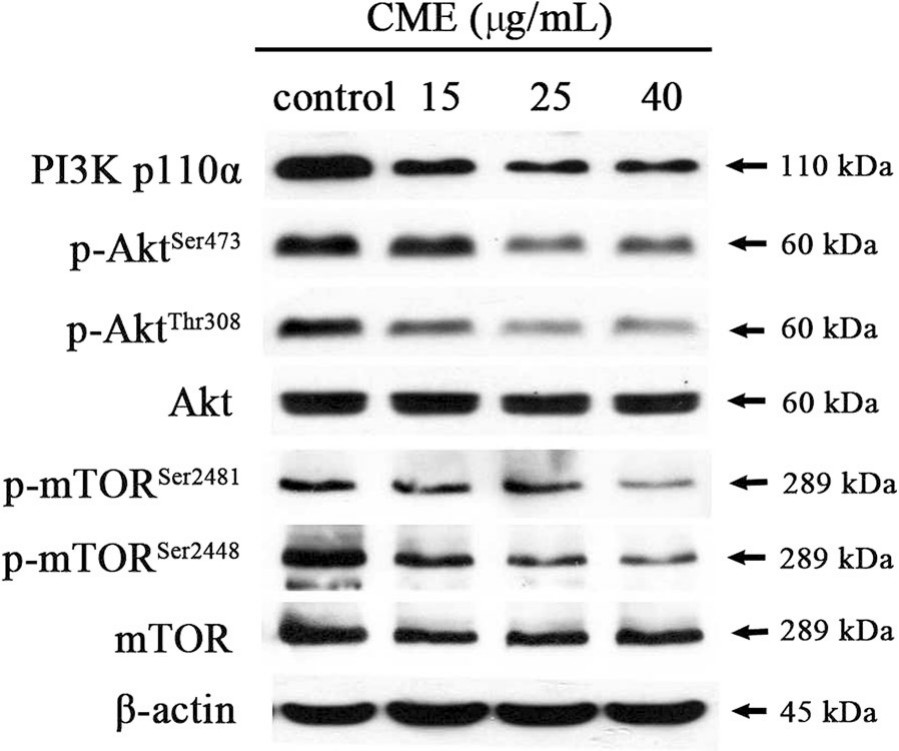
Inhibition of the PI3K-Akt-mTOR signaling pathway in CNE-1 cells by CME. After treatment with the indicated concentrations of CME for 24 h, cell lysates were subjected to western blot analysis using the indicated antibodies against PI3K p110α, Akt and p-Akt (Thr^308^ and Ser^473^), mTOR and p-mTOR (Ser^2448^ and Ser^2481^). β-actin served as a loading control

We investigated whether CME treatment could also down-regulate the expression levels of related proteins in the PI3K-Akt-mTOR pathway. After treatment with CME (15, 25, 40 μg/mL) for 24 h, the levels of PI3Kinase P110α, the catalytic subunit of PI3K (*P* = 0.042, 0.040, 0.030), and the amount of phosphorylation of Akt at Ser^473^ (*P* = 0.047, 0.0008, 0.0007) and Thr^308^ (*P* = 0.021, 0.005, 0.004) were remarkably decreased in a concentration-dependent manner, while the amount of total Akt remained constant following CME treatment (Fig. 6). Consistently, phosphorylation of the downstream effector mTOR at Ser^2448^ (*P* = 0.037, 0.025, 0.006) and Ser^2481^ (*P* = 0.046, 0.039, 0.022) also showed a dose-dependent decrease, and the amount of total mTOR was slightly down-regulated upon CME treatment of CNE-1 cells.

## Discussion

In the present study, the anti-cancer effects of CME toward human nasopharyngeal carcinoma CNE-1 cells in terms of cytotoxicity, apoptosis induction and cell cycle arrest were investigated. CME possessed significant cytotoxic activities against CNE-1 cells, in a time- and dose-dependent manner, but very low cytotoxicity toward normal LO2 cells. These results suggest that CME has potential selective anti-cancer effects on CNE-1 cells.

Apoptotic induction in cancer cells is a crucial therapeutic strategy for cancer treatment. CME induced morphological changes including cell shrinkage, the appearance of apoptotic vacuoles, and chromatin condensation, detected by fluorescence microscopy after CME treatment followed by Ho 35288 staining. This apoptotic induction was confirmed by flow cytometry using an annexin V-FITC/PI double-staining approach.

In this study, changes in cell cycle progression were analyzed by flow cytometry. CME induced obvious G2/M phase arrest in CNE-1 cells in a dose- and time-dependent manner. G2/M, the most important checkpoint for DNA damage, is critical to cell cycle progression, and strictly regulated by specific genes [21]. If phase arrest cannot be repaired, cells may undergo apoptosis.

Changes in the expression levels of apoptosis-related proteins in CNE-1 cells after CME treatment were evaluated by western blotting to explore the mechanism underlying the induction of apoptosis by CME in CNE-1 cells. The activation of caspase 9 through apoptotic protease activating factor-1 (APAF-1) and cytochrome c release [22] initiates activation of the caspase cascade, cleaves executioner caspase-3, and eventually results in apoptosis [23]. Cleaved caspase 8 activates downstream caspases such as caspase-3, -6, and -7 [24], which cleave specific intracellular substrates, such as polyADP-ribose polymerase (PARP), lamins and inhibitor of caspase activated DNase (ICAD), finally resulting in programmed cell death [25]. This study revealed that CME could significantly induce CNE-1 cell apoptosis via the activation of caspase-8, -9, -3, as well as PARP, through both extrinsic and intrinsic pathways.

The B cell lymphoma 2 (Bcl-2) family proteins, including pro-apoptotic members such as Bax, Bak and anti-apoptotic members such as Bcl-2, Bcl-xL, play important roles in the regulation of mitochondrial-mediated apoptosis [26, 27]. In this study, CME treatments downregulated the expression of Bcl-2, and upregulated the expression of Bax, resulting in CNE-1 cell apoptosis. Furthermore, elevated intracellular ratios of Bax/Bcl-2 were observed during a period of increased apoptotic cell death. These experimental findings suggested that CME induced CNE-1 cell apoptosis via a mitochondrial pathway.

**Fig. 7:**
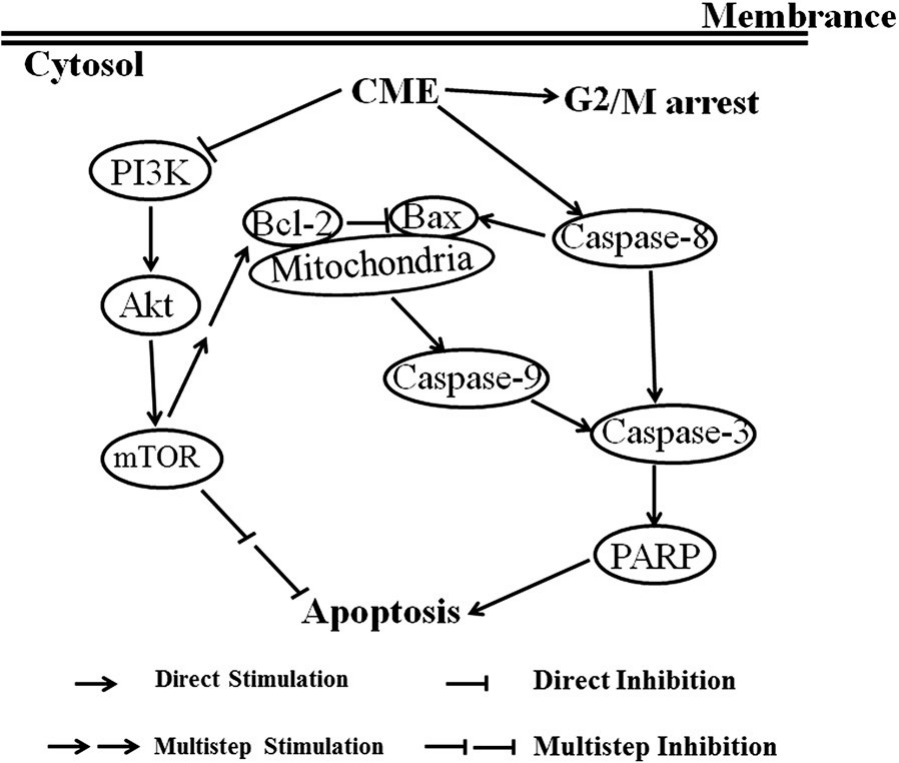
Schematic diagram showing the hypothetical effects of CME on the PI3K-Akt-mTOR/signaling pathway and apoptosis in CNE-1 cells

The PI3K-AKT-mTOR pathway is a major signaling pathway in cancer [28]. This pathway is frequently mutated and overactivated in human cancers [29]. The PI3K-AKT-mTOR pathway promotes survival through inhibition of proapoptotic factors and activation of anti-apoptotic factors [30]. Through phosphorylation of pathway components, this pathway inhibits the activity of proapoptotic members while activating anti-apoptotic members [31]. Therefore, blockade of the PI3K-AKT-mTOR pathway has become a cancer therapy strategy. Our present results indicate for the first time that CME downregulates the PI3K-AKT-mTOR-mediated signaling in CNE-1 cells (Fig. 7). Additionally, 14-thienyl methylene matrine (YYJ18), a derivative of matrine, has been reported to induce apoptosis in NPC cells by targeting PI3K-Akt [32], which also provides evidence for the involvement of this pathway in NPC carcinogenesis.

## Conclusion

CME inhibited the proliferation of CNE-1 cells and activation of the PI3K-AKT-mTOR signaling pathway.

## Abbreviations

CME: *Centipeda minma* ethanol extracts
CM: Chinese medicine
CNE-1: high differentiated human nasopharyngeal carcinoma cells
CNE-2: low differentiated human nasopharyngeal carcinoma cells
LO2: human hepatocytes
NPC: nasopharyngeal carcinoma
MTT: 3-(4,5-dimethylthiazole-2-yl)-2,5-diphenyltetrazolium bromide
DMSO: dimethyl sulfoxide
FBS: fetal bovine serum
RPMI1640: Roswell Park Memorial Institute1640 medium
PBS: phosphate buffered saline
PI: peromide iodine
FITC: fluorescein-isothiocyanate
SDS: sodium dodeeyl sulphate
PAGE: polyacrylamidegel electrophoresis
T-TBS: tween-20
PVDF: polyrinylidene difluoride
RNase A: ribonuclease A
ICAD: lamins and inhibitor of caspase activated DNase
PARP: polyADP-ribose polymerase
PI3K: phosphatidylinositol 3-kinase
Akt: protein kinase B
mTOR: the mammalian target of rapamycin
Bcl-2: B cell lymphoma/lewkmia-2
Bax: Bcl-2 assaciated X protein
YYJ18: 14-thienyl methylene matrine (YYJ18)
Caspase: cysteinyl aspartate specific proteinase
